# Characterizing Musculoskeletal Sequelae in Ebola Virus Survivors During the 7 Years Since Hospital Discharge in Eastern Sierra Leone

**DOI:** 10.1093/ofid/ofaf129

**Published:** 2025-03-08

**Authors:** Anna C Sanford, Nell G Bond, Emily J Engel, Foday Alhasan, Michael Gbakie, Fatima Kamara, Lansana Kanneh, Ibrahim Mustapha, Mohamed Yillah, Donald Grant, Robert Samuels, John S Schieffelin

**Affiliations:** Tulane University, New Orleans, Louisiana, USA; Tulane University, New Orleans, Louisiana, USA; Tulane University, New Orleans, Louisiana, USA; Kenema Government Hospital, Kenema, Sierra Leone; Kenema Government Hospital, Kenema, Sierra Leone; Kenema Government Hospital, Kenema, Sierra Leone; Kenema Government Hospital, Kenema, Sierra Leone; Kenema Government Hospital, Kenema, Sierra Leone; Kenema Government Hospital, Kenema, Sierra Leone; Kenema Government Hospital, Kenema, Sierra Leone; Tulane University, New Orleans, Louisiana, USA; Tulane University, New Orleans, Louisiana, USA

**Keywords:** Ebola virus disease, post-Ebola syndrome, post-viral sequelae, musculoskeletal

## Abstract

Ebola virus disease survivors demonstrated musculoskeletal sequelae during the 7 years since hospital discharge. Reported joint pain and joint tenderness to palpation were the most common sequelae. Sequelae generally decreased over time, but fluctuations were noted. Survivors 15–40 years of age demonstrated the highest rates of sequelae throughout study duration.

Ebola virus disease (EVD) survivors present with heterogeneous post-Ebola sequelae described as post-Ebola syndrome (PES) [1–3]. Musculoskeletal (MSK) sequelae, including muscle and joint pain, are some of the most common complaints in EVD survivors with PES [2–4]. Longitudinal studies of PES are necessary for understanding and management of sequelae. As targeted treatments for Ebola virus develop, the number of EVD survivors will likely increase with subsequent outbreaks. Without further characterization of PES over time, our ability to identify and treat EVD sequelae is limited.

Our group previously demonstrated a phenotype of PES symptoms based on the presence of MSK sequelae, suggesting that survivors with MSK sequelae may require specific treatment [3]. This MSK phenotype was associated with abdominal tenderness [3]. Previous studies followed up survivors only for the first few years after EVD [2], or reported symptoms without physical examination findings [5, 6]. We hypothesized that MSK sequelae in EVD survivors would follow the arthralgia pattern demonstrated after infection with Chikungunya virus and coronavirus disease 2019 (COVID-19), with higher rates in those who were female or in older age groups [7, 8], and that sequelae would decrease over time. Here, we present an analysis of MSK signs and symptoms in EVD survivors over a 7-year period after discharge from the Ebola treatment unit (ETU).

## METHODS

This study was conducted as part of an ongoing cohort study following EVD survivors and their household contacts in Sierra Leone. Data were collected between March 2016 and March 2022. Reported “MSK symptoms” include current joint or muscle pain. We included abdominal tenderness to palpation with MSK physical examination signs (decreased joint range of motion, joint tenderness to palpation, and joint edema and/or effusion) due to the association identified by agglomerative clustering [3].

Data were analyzed using R Studio with R software, version 4.2.2. Logistic regression was used to determine variables associated with survivor status and MSK sequelae at enrollment. Time from ETU discharge to visit, continuous age, and sex were controlled for in regressions when applicable. A χ^2^ analysis was used for pairwise comparisons of unadjusted odds ratios at first visit. The MFX package was used to determine adjusted odds ratios at the first visit (v 1.2-2) [9]. Cumulative incidence graphs were created using the tidycmprsk package (v 0.2.0) [10]. The GLMMadaptive package was used to create generalized linear mixed models (v 0.9-1) [11]. For a complete description of methods used in this study, please see the Supplementary Methods.

### Institutional Review Board Determination and Written Consent

This study was approved by the Tulane University Institutional Review Board (approval no. 701226) and the Sierra Leone Ethics and Scientific Review Committee. We obtained written informed consent from adult participants (aged ≥18 years), consent by a parent or guardian and assent in children 12–17 years old, and consent by a parent or guardian in children <12 years old. Study personnel were all trained in ethics and research compliance.

## RESULTS

### Demographics of EVD Survivors and Household Contacts

Between March 2016 and September 2019, a total of 379 survivors and 1040 contacts were enrolled. Participants completed questionnaires and physical examinations and were followed up through March 2022. At enrollment, the median age of survivors was significantly higher than that of contacts (29 vs 19 years; *P* < .001), and more survivors than contacts were female, although this was not significant (*P* = .09; Supplementary Table 1). Dates of discharge ranged from May 2014 to January 2016, and the median date of discharge was 30 August 2014.

### MSK Sequelae at Enrollment

At enrollment, survivors were more likely than household contacts to demonstrate MSK signs at physical examination (23.8% vs 7.5%; *P* < .001) and report current MSK symptoms (38.9% vs 9.0%; *P* < .001; Supplementary Table 1). At enrollment, 38.4% and 23.8% of survivors reported current joint and muscle pain, respectively. The most demonstrated physical examination findings at enrollment were joint tenderness to palpation (17%), abdominal tenderness (11.1%), decreased joint range of motion (10.6%), and joint edema and/or effusion (2.1%).

Survivors with a shorter time from discharge to first visit were more likely to present with MSK signs or symptoms (*P* < .001; Supplementary Table 2). Survivors reporting MSK issues before EVD diagnosis were more likely to present with MSK symptoms (*P* < .001) but not signs (*P* = .44). Reporting depression predicted current MSK symptoms (*P* < .001) but not signs (*P* = .68). No individual occupation was significant for predicting MSK signs or symptoms when controlling for age, sex, and time from discharge (data not shown).

When the analysis included continuous age at enrollment, survivors reporting MSK symptoms were significantly older than those without MSK symptoms (*P* = .009; Supplementary Table 2). However, continuous age did not predict MSK physical examination signs at enrollment. While more survivors than contacts reported MSK symptoms in all age categories and survivors were more likely to demonstrate MSK signs at physical examination in most age categories, there was no significant difference in MSK signs between survivors and contacts >40 years of age (16.0% vs 12.0%; *P* = .42; Supplementary Table 3). The highest rates of MSK sequelae were observed in survivors 15–40 years old. Approximately 74% of survivors with MSK signs and/or symptoms were in the 15–40-year age group. Survivors aged 15–40 years had 3.1 times higher odds of demonstrating MSK signs at first physical examination compared to survivors <15 years old (*P* = .02; Supplementary Figure 1 and Supplementary Table 4). Survivors in the 15–40-year and >40-year groups had higher odds of reporting MSK symptoms than those <15 years old (*P* < .001).

### MSK Sequelae Over Time

We followed up survivors for up to 7.8 years after ETU discharge (444–2850 days; Supplementary Figure 2 and Supplementary Table 5). Six survivors presented for follow-up in year 7 after ETU discharge and were grouped with survivors from year 8. Of the 366 survivors included in longitudinal analysis, 1133 total visits were completed, with an average of 3 visits per survivor. Approximately 77% of survivors reported current joint and/or muscle pain within the earliest time point of our study—2 years after ETU discharge. The rate of joint and/or muscle pain decreased to 13.8% at 7–8 years after ETU discharge. Joint pain was reported more often than muscle pain in all years. While sequelae generally decreased over time, survivors reported MSK symptoms at a higher rate in years 5 and 6 compared with the prior year (difference not significant).

**Figure 1.**
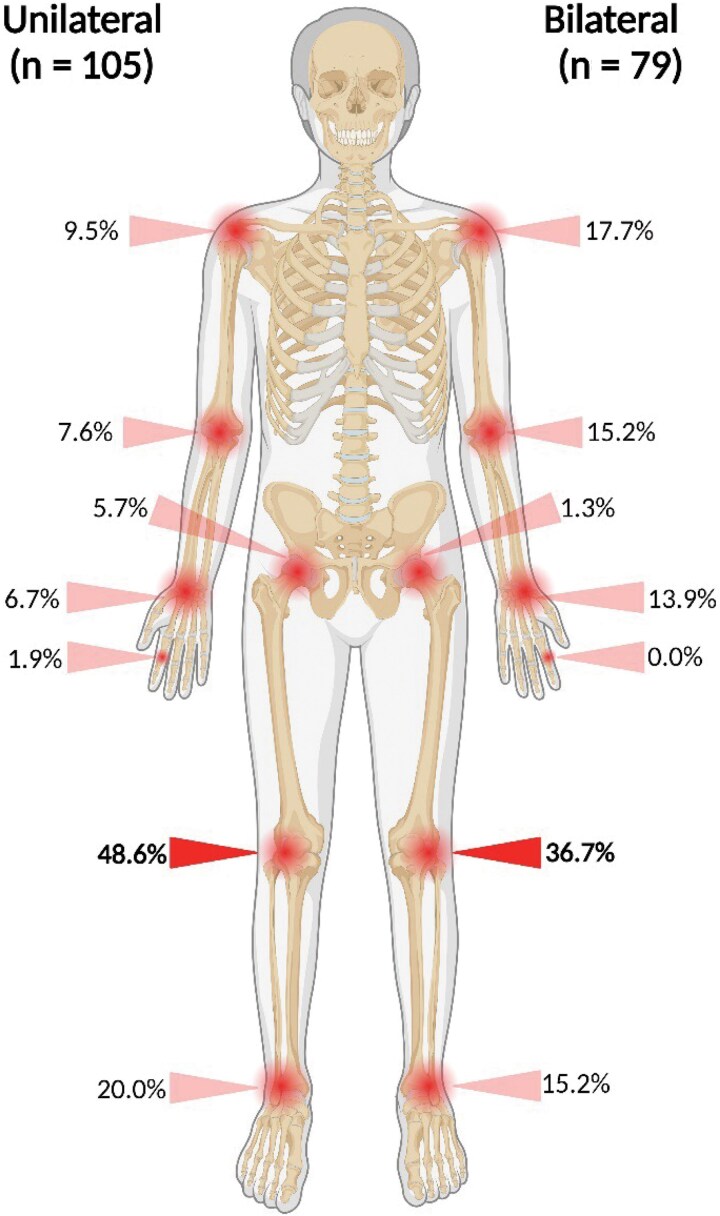
Percentages of musculoskeletal (MSK) signs by location at any point since discharge from the Ebola treatment unit. MSK signs occur at numerous locations but most often unilaterally at the knee. (Figure created in BioRender; https://BioRender.com/g96q793.)

Joint signs also decreased over time. During year 2 since ETU discharge, 43.4% of survivors demonstrated ≥1 MSK examination sign, decreasing to 4.4% by years 7–8. Joint tenderness to palpation was the most noted examination sign (Supplementary Figure 2 and Supplementary Table 5). Survivors were most likely to demonstrate MSK signs in the knee (Figure 1 and Supplementary Table 6). The most demonstrated signs, joint tenderness to palpation and decreased range of motion, were bilateral 43.3% and 41.3% of the time, respectively.

**Figure 2.**
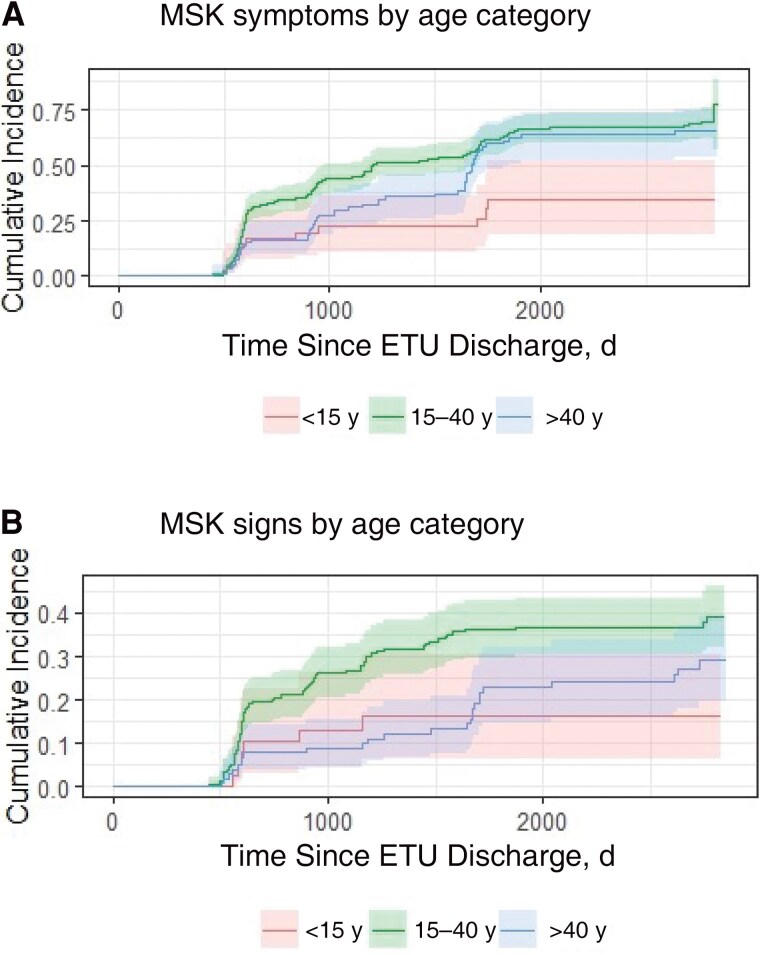
Cumulative incidence of musculoskeletal (MSK) sequelae in Ebola virus disease survivors by age category over the duration of the study in Eastern Sierra Leone. Cumulative incidence of MSK symptoms and signs was highest in the 15–40-year age group throughout the study duration. *A,* Cumulative incidence of MSK symptoms. Survivors in the 15–40-year age group demonstrate the highest cumulative incidence. *B,* Cumulative incidence of MSK signs. As with MSK symptoms, survivors 15–40 years of age demonstrated the highest cumulative incidence. Age categories were adjusted for participant age at the time of observation. Abbreviation: ETU, Ebola treatment unit. (Graph made in R Studio version 4.2.2.)

In total, 220 EVD survivors (60.1%) reported MSK symptoms, and 115 (31.4%) exhibited MSK signs on examination at least once during study duration. The incidence of MSK signs and symptoms increased more sharply and remained highest in the 15–40-year age group (Figure 2). The cumulative incidence of MSK sequelae in the >40-year age group increased after the halfway point of the study but remained below that in survivors 15–40 years of age.

A generalized linear mixed model determined predictors of MSK sequelae in EVD survivors over the study duration (Supplementary Table 7). Continuous age was positively correlated with MSK symptoms (*P* < .001) but not signs (*P* = .06). A shorter time from ETU discharge to visit was also correlated with MSK symptoms and signs (both *P* < .001). Compared with survivors aged <15 years, those aged 15–40 years had significantly higher odds of reporting MSK symptoms but not demonstrating MSK signs. Age 15–40 versus >40 years was significant for predicting MSK signs, but not symptoms. Female sex did not predict MSK symptoms or signs. Self-reported depression predicted MSK symptoms but not signs.

## DISCUSSION

We provide a comprehensive report of MSK sequelae of PES for >7 years since ETU discharge. Most longitudinal studies of EVD survivors follow reported symptoms rather than examination findings [5, 6]. By including examination findings, we present a more objective and comprehensive view of MSK sequelae in PES. To our knowledge, there have been no studies focused on the influence of age on MSK or other system-grouped sequelae in EVD survivors.

Arthralgias are the most common MSK symptom in EVD survivors [12, 13]. This was true in our study, as joint pain was the most common MSK complaint. MSK symptoms fluctuated more in our study than in prior studies, where a steady annual decline was observed [5]. This could be due to a waxing and waning of symptoms or different perceptions of symptom presence over time. MSK signs may be more reliable data markers of MSK sequelae than self-reported MSK symptoms due to the risk of recall bias inherent in self-reporting symptoms.

Risk factors for rheumatic sequelae due to other viral infections (Chikungunya virus and COVID-19) include female sex and age >45 years [7, 8]. Our study found the highest rates of MSK sequelae in survivors 15–40 years old. Possible explanations for the increase in MSK sequelae in this age group include accelerated joint aging in this younger group due to EVD effects, inflammatory response to EVD, or increased viral proliferation in joint spaces. Further studies are needed to elucidate the mechanism of EVD sequelae development, including the role of age in immune response to EVD infection.

## Supplementary Material

ofaf129_Supplementary_Data

## Supplementary Data

Supplementary materials are available at *Open Forum Infectious Diseases* online. Consisting of data provided by the authors to benefit the reader, the posted materials are not copyedited and are the sole responsibility of the authors, so questions or comments should be addressed to the corresponding author.
