# Supplementary material for: Characterizing Musculoskeletal Sequelae in Ebola Virus Survivors During the 7 Years Since Hospital Discharge in Eastern Sierra Leone
Source: Open Forum Infect Dis. 2025 Mar 8;12(4):ofaf129. doi: 10.1093/ofid/ofaf129 (PMC11952999; doi:10.1093/ofid/ofaf129)
Supplement: ofaf129_Supplementary_Data [file ofaf129_supplementary_data.zip › MSK_supplemental_2_25.docx]

**Characterizing Musculoskeletal Sequelae in Ebola Virus Survivors Over Seven Years Since Hospital Discharge in Eastern Sierra Leone: Supplemental Data**

**Supplemental Methods**

***Study Design & Data Collection***

Data was collected between March 2016-March 2022 as part of an ongoing cohort study following EVD survivors and their household contacts previously described by Bond, et. al.^1^ Participants were identified using an EVD survivor registry maintained by the Sierra Leone Association of Ebola Survivors (SLAES). Participants answered a symptom questionnaire and underwent a physical exam at each visit. Follow-up visits were scheduled approximately every six months. However, some visits could not be completed due to the COVID-19 pandemic. Demographic data, collected during the enrollment visit, was previously described.^1^

Only participants who completed a questionnaire and physical exam were included in this analysis. Contacts were not required to be Ebolavirus (EBOV) seronegative and therefore were not a true independent control group. Information on household contact selection was previously described.^1^ Participants were eligible if they were at least seven years of age. Additionally, survivors with self-reported MSK symptoms before diagnosis were included in this study to evaluate whether EVD could worsen pre-existing symptoms. Survivors were defined as persons with a positive test for EBOV at hospital admission and certificate of hospital discharge. Vaccination against Ebola and Ebola-specific treatments were not available during the illness course of survivors in our study.

***Questionnaire and Physical Exam***

Survivors and household contacts responded to the same questionnaire and underwent the same physical exams. One survivor and one contact did not provide information on age and sex and were excluded when these variables were analyzed. 369 (97%) survivors provided information on date of hospital discharge and first visit. Three survivors reported dates of discharge before or after the accepted outbreak period. These survivors were excluded when date of discharge was included in analysis. Three-hundred and sixty-two survivors provided information on occupation.

Reported symptoms considered “MSK symptoms” from the questionnaire include current joint or muscle pain. Only musculoskeletal symptoms occurring at the time of the visit were included. Symptoms associated with depression include reporting depression at the time of the visit. In the event responses to specific symptoms were not recorded, they were considered not present.

We previously found EVD survivors in our cohort clustered into three distinct phenotypes: no sequelae, non-MSK sequelae, and primarily MSK sequelae.^1^ The MSK phenotype, particularly joint tenderness to palpation and decreased joint range of motion, was associated with abdominal tenderness (any quadrant). The relationship between MSK sequelae and abdominal tenderness was identified using the Ward method in agglomerative clustering in our prior work. Therefore, we included abdominal tenderness to palpation with MSK physical exam signs due to the close clustering. Depression was also associated with MSK sequelae and was analyzed separately. Physical exam findings considered positive for MSK signs include decreased or absent joint range of motion, joint tenderness to palpation, joint edema and/or effusion, and abdominal tenderness. If MSK signs were not recorded by the provider performing the exam, the participant was considered to not have MSK signs.

***Data Analysis***

Data was analyzed using R Studio with R version 4.2.2 (Boston, MA). Logistic regression was used to determine which variables were associated with survivor status and MSK sequelae at enrollment. Time from ETU discharge to visit, continuous age and sex were controlled for in regressions when applicable. Chi square analysis was used for pairwise comparisons of unadjusted odds ratios at first visit. The MFX package was used to determine adjusted odds ratios and significance at first visit.^2^ Cumulative incidence graphs were created using the tidycmprsk package.^3^ The GLMMadaptive package was used to create generalized linear mixed models (GLMM).^4^ GLMMs were used to determine significance of several factors, including age category, for predicting MSK sequelae over time. These models included continuous age (except when analyzing significance of age categories), sex, and time from discharge to visit as well as interaction between the effect of interest and time as fixed effects. Participant ID was included as the random effect in GLMMs. Graphics were created in R, GraphPad Prism version 9.2.0 (San Diego, CA), and BioRender.

Survivors and contacts were grouped by age at enrollment: <15 years of age, 15-40 years, and >40 years. These age categories were chosen to analyze whether reproductive age influences MSK sequelae and were derived from female fertility data from the Multiple Indicator Cluster Survey 2017 in Sierra Leone, which demonstrated a sharp decrease in fertility around age 40.^5^ Males and females were grouped by the same age categories.

**References**

1. Bond NG, Grant DS, Himmelfarb ST, et al. Post-Ebola syndrome presents with multiple overlapping symptom clusters: evidence from an ongoing cohort study in Eastern Sierra Leone. *Clin Infect Dis*. Apr 03 2021;doi:10.1093/cid/ciab267

2. *mfx: Marginal Effects, Odds Ratios and Incidence Rate Ratios for GLMs*. 2019.

3. *tidycmprsk: Competing Risks Estimation*. 2023.

4. *GLMMadaptive: Generalized Linear Mixed Models using Adaptive Gaussian Quadrature*. 2023.

5. *Sierra Leone Multiple Indicator Cluster Survey 2017 Survey Findings Report*. 2017:77. <https://mics-surveys-prod.s3.amazonaws.com/MICS6/West%20and%20Central%20Africa/Sierra%20Leone/2017/Survey%20findings/Sierra%20Leone%202017%20MICS%20Survey%20Findings%20Report_English.pdf>

**Supplementary Tables and Figures**

**Supplementary Table 1: Demographics of EVD Survivors and Household Contacts at First Visit Since EVD Outbreak in Eastern Sierra Leone**

| **Characteristic** | **Survivors (n=379)** | **Contacts (n=1040)** | **p-value** |
| --- | --- | --- | --- |
| Age (years), median, (Q1-Q3) | 29 (18-39) | 19 (15-28) | <.001 |
| Female sex, n, (%) | 205 (54.2) | 491 (47.3) | .086 |
| Days of hospitalization, median, (Q1-Q3) | 22 (14-30) | NA | - |
| Days from discharge to first visit, median, (Q1-Q3) | 948 (593-1201) | NA | - |
| MSK symptoms, n, (%) | 147 (38.9) | 94 (9.0) | <.001 |
| MSK signs, n, (%) | 90 (23.8) | 78 (7.5) | <.001 |
| MSK signs and/or symptoms, n, (%) | 176 (46.6) | 158 (15.2) | <.001 |

Survivors were significantly older than household contacts and more likely to demonstrate MSK sequelae. Logistic regression performed in R Studio and adjusted for age and sex. Abbreviations: Q1-Q3: 1^st^ to 3^rd^ quartile, MSK: musculoskeletal.

**Supplementary Table 2: Demographics of EVD Survivors With and Without MSK Sequelae at Enrollment**

| **Characteristic** | **Survivors w/ MSK Symptoms (n=147)** | **Survivors w/o MSK Symptoms (n=232)** | **p-value** | **Survivors w/ MSK Signs**  **(n= 90)** | **Survivors w/o MSK Signs**  **(n= 289)** | **p-value** | **Survivors w/ MSK Symptoms and/or Signs (n=176)** | **Survivors w/o MSK Symptoms and/or Signs (n=203)** | **p-value** |
| --- | --- | --- | --- | --- | --- | --- | --- | --- | --- |
| Age (years), median, (Q1-Q3) | 31 (22-38) | 26 (16-40) | .009 | 29.5 (20-36) | 29 (18-40) | .891 | 30 (21-38) | 26.5 (15-40) | .042 |
| Age < 15 years, n, % | 9 (6.1) | 50 (21.6) | ref | 7 (7.8) | 52 (18.0) | ref | 12 (6.8) | 47 (23.3) | ref |
| Age 15-40 years, n, % | 109 (74.1) | 129 (55.8) | <.001 | 70 (77.8) | 168 (58.1) | .017 | 130 (73.9) | 108 (54.5) | <.001 |
| Age > 40 years, n, % | 29 (19.7) | 52 (22.5) | <.001 | 13 (14.4) | 68 (23.5) | .361 | 34 (19.3) | 47 (23.3) | <.001 |
| Female sex, n, (%) | 88 (59.9) | 117 (50.6) | .363 | 51 (56.7) | 154 (53.3) | .954 | 101 (57.4) | 104 (51.5) | .706 |
| Depression, n, (%) | 42 (28.6) | 5 (2.2) | <.001 | 16 (17.8) | 31 (10.8) | .680 | 42 (23.9) | 5 (2.5) | <.001 |
| Days of hospitalization, median, (Q1-Q3) | 20 (14-30) | 26 (17-30) | .429 | 21 (14-30) | 23 (14-30) | .716 | 21 (14-30) | 27 (16-30) | .369 |
| Days from discharge to first visit, median, (Q1-Q3) | 599 (563-903) | 1173 (932-1219) | <.001 | 600 (568-899) | 1154 (676-1209) | <.001 | 603 (567-933) | 1179 (948-1222) | <.001 |
| MSK symptoms before EVD diagnosis, n, (%) | 42 (28.6) | 7 (3.0) | <.001 | 19 (21.1) | 30 (10.4) | .442 | 43 (24.4) | 6 (3.0) | <.001 |
| MSK symptoms since EVD diagnosis, n, (%) | 144 (98.0) | 19 (8.2) | <.001 | 64 (71.1) | 99 (34.3) | .073 | 147 (83.5) | 16 (7.9) | <.001 |
| Current MSK symptoms, n, (%) | 147 (100.0) | NA | - | 61 (67.8) | 86 (29.8) | .008 | 147 (83.5) | NA | - |

Age category and time from hospital discharge to first visit can predict MSK sequelae to varying degrees. Logistic regression performed in R Studio and adjusted for age, sex, and time from discharge to visit. Abbreviations: Q1-Q3: 1^st^ to 3^rd^ quartile, MSK: musculoskeletal, EVD: Ebola virus disease.

**Supplementary Table 3: MSK Sequelae in EVD Survivors and Household Contacts by Age Category at Enrollment**

| **Characteristic** | **Survivors (n=378)** | **Contacts (n=1039)** | **p-value** |
| --- | --- | --- | --- |
| Age Categories | | | |
| Age < 15 years, n, % | 59/378 (15.6) | 254/1039 (24.4) | ref* |
| Age 15-40 years, n, % | 238/378 (63.0) | 693/1039 (66.7) | .020 |
| Age > 40 years, n, % | 81/378 (21.4) | 92/1039 (8.9) | <.001 |
| Female Age Categories | | | |
| Age <15 years, n, % | 34/205 (16.6) | 106/491 (21.6) | ref* |
| Age 15-40 years, n, % | 129/205 (62.9) | 336/491 (68.4) | .419 |
| Age >40 years, n, % | 42/205 (20.5) | 49/491 (10.0) | <.001 |
| MSK Symptoms | | | |
| n/N, (%) | 147/378 (38.9) | 94/1039 (9.1) | <.001 |
| < 15 years | 9/59 (15.3) | 10/254 (3.9) | .001 |
| 15-40 years | 109/238 (45.8) | 68/693 (9.8) | <.001 |
| >40 years | 29/81 (35.8) | 16/92 (17.4) | .009 |
| MSK Signs | | | |
| n/N, (%) | 90/378 (23.8) | 78/1039 (7.5) | <.001 |
| < 15 years | 7/59 (11.9) | 7/254 (2.8) | .004 |
| 15-40 years | 70/238 (29.4) | 60/693 (8.9) | <.001 |
| >40 years | 13/81 (16.0) | 11/92 (12.0) | .419 |
| MSK Signs and/or Symptoms | | | |
| n/N, (%) | 176/378 (46.6) | 158/1039 (15.2) | <.001 |
| < 15 years | 12/59 (20.3) | 16/254 (6.3) | .001 |
| 15-40 years | 130/238 (54.6) | 119/693 (17.2) | <.001 |
| >40 years | 34/81 (42.0) | 23/92 (25.0) | .029 |

More survivors than contacts were in the >40 age group, and more contacts than survivors were in the 15-40 age group. Survivors were significantly more likely to demonstrate MSK sequelae than contacts, except when comparing MSK signs in survivors and contacts > 40 years. For predicting age categories and female age categories in survivors vs contacts, age <15 years was the reference age category. Logistic regression performed in R Studio and adjusted for female sex. Abbreviations: MSK= musculoskeletal, EVD= Ebola virus disease

**Supplementary Table 4: Adjusted and Unadjusted Odds of MSK Sequelae in EVD Survivors at Enrollment**

| **MSK Symptoms** | | | | |
| --- | --- | --- | --- | --- |
| Age Categories | Unadjusted OR [CI] | p-value | Adjusted OR [CI] | p-value |
| 15-40 vs <15 y | 4.6 [2.3, 10.5] | <.001 | 7.5 [3.1, 20.1] | <.001 |
| 15-40 vs > 40 y | 1.5 [0.9, 2.6] | .117 | 1.1 [0.6, 2.2] | .715 |
| >40 vs <15 y | 3.0 [1.4, 7.5] | .007 | 6.6 [2.4, 19.9] | <.001 |
| **MSK Signs** | | | | |
| Age Categories | Unadjusted OR [CI] | p-value | Adjusted OR [CI] | p-value |
| 15-40 vs <15 y | 3.0 [1.4, 7.7] | .006 | 3.1 [1.3, 8.8] | .017 |
| 15-40 vs > 40 y | 2.2 [1.2, 4.3] | .018 | 1.9 [0.9, 4.0] | .090 |
| >40 vs <15 y | 1.4 [0.5, 4.0] | .485 | 1.7 [0.6, 5.3] | .361 |
| **MSK Signs and/or Symptoms** | | | | |
| Age Categories | Unadjusted OR [CI] | p-value | Adjusted OR [CI] | p-value |
| 15-40 vs <15 y | 4.7 [2.4, 9.6] | <.001 | 6.7 [3.0, 16.3] | <.001 |
| 15-40 vs > 40 y | 1.7 [1.0, 2.8] | .049 | 1.3 [0.7, 2.4] | .474 |
| >40 vs <15 y | 2.8 [1.3, 6.3] | .007 | 5.3 [2.1, 14.4] | <.001 |

Adjusted odds ratios are adjusted for sex and time from hospital discharge to first visit. Statistics performed in R Studio. Abbreviations: y= years, MSK= musculoskeletal, EVD= Ebola virus disease

**Supplementary Table 5: MSK Signs and Symptoms in EVD Survivors More Than Seven Years Since ETU Discharge in Eastern Sierra Leone**

| **Year (days since ETU discharge)** | **Year 2 (444-731)** | **Year 3 (732-1097)** | **Year 4 (1098-1463)** | **Year 5 (1464-1829)** | **Year 6 (1830-2195)** | **Year 7-8 (2196-2850)** |
| --- | --- | --- | --- | --- | --- | --- |
| **Reported symptoms, n/N, (%)** | | | | | | |
| Joint Pain | 96/127 (75.6) | 85/163 (52.1) | 36/204 (17.6) | 103/283 (36.4) | 45/123 (36.6) | 20/190 (10.5) |
| Muscle Pain | 59/127 (46.5) | 49/160 (30.6) | 15/203 (7.4) | 43/283 (15.2) | 24/122 (19.7) | 10/192 (5.2) |
| 1+ MSK symptom, n/N, (%) | 98/127 (77.2) | 86/164 (52.4) | 36/204 (17.6) | 108/283 (38.2) | 48/123 (39.0) | 27/196 (13.8) |
| **Physical exam signs, n/N, (%)** | | | | | | |
| 1+ joint decreased ROM | 37/129 (28.7) | 4/164 (2.4) | 5/204 (2.5) | 9/283 (3.2) | 1/123 (0.8) | 6/205 (2.9) |
| Edema or effusion | 6/129 (4.7) | 4/164 (2.4) | 1/204 (0.5) | 1/283 (0.4) | 0/123 (0.0) | 0/205 (0.0) |
| Joint tender to palpation | 42/129 (32.6) | 11/164 (6.7) | 15/204 (7.4) | 10/283 (3.5) | 1/123 (0.8) | 4/205 (2.0) |
| Abdominal tenderness | 30/129 (23.3) | 13/164 (7.9) | 4/204 (2.0) | 12/283 (4.2) | 1/123 (0.8) | 2/205 (1.0) |
| 1+ MSK PE sign | 56/129 (43.4) | 25/164 (15.2) | 19/204 (9.3) | 23/283 (8.1) | 3/123 (2.4) | 9/205 (4.4) |
| 1+ MSK sign and/or symptom, n/N, (%) | 104/129 (80.6) | 98/164 (59.8) | 50/204 (24.5) | 114/283 (40.3) | 50/123 (40.7) | 31/205 (15.1) |

Corresponding table to heatmap (Figure 2). Statistics performed in R Studio. Abbreviations: MSK= musculoskeletal, EVD= Ebola virus disease, ETU= Ebola treatment unit

**Supplementary Table 6: MSK Signs and Locations at Any Visit Since ETU Discharge**

| **MSK Sign** | **Location** | **% Bilateral Joints, (n/N)** |
| --- | --- | --- |
| 1+ joint decreased ROM (n=63) | \| Hip (n=4) \| \| --- \| \| Knee (n=34) \| \| Ankle (n=15) \| \| Shoulder (n=9) \| \| Elbow (n=6) \| \| Wrist (n=6) \| \| Interphalangeal (n=1) \| | 41.3, (31/75) |
| Edema or effusion (n=13) | \| Knee (n=5) \| \| --- \| | 60.0, (3/5) |
| Joint tenderness to palpation (n=86) | \| Hip (n=3) \| \| --- \| \| Knee (n=41) \| \| Ankle (n=18) \| \| Shoulder (n=15) \| \| Elbow (n=14) \| \| Wrist (n=12) \| \| Interphalangeal (n=1) \| | 43.3, (45/104) |
| Abdominal tenderness (n=63) | \| Suprapubic (n=12) \| \| --- \| \| Periumbilical (n=3) \| \| Generalized (n=2) \| \| RUQ (n=10) \| \| RLQ (n=8) \| \| LUQ (n=7) \| \| LLQ (n=10) \| | NA |

The left column describes the number of survivors with specific musculoskeletal sequelae at any time during the study period. Not all signs had documented locations, therefore the sum of middle column signs with locations does not equal the “n” in the left column in all cases. For percent bilateral joints, the denominator used was the sum of all defined locations (middle column). Statistics performed in Microsoft Excel. Abbreviations: MSK= musculoskeletal, ETU= Ebola treatment unit, ROM= range of motion, RUQ= right upper quadrant, RLQ= right lower quadrant, LUQ= left upper quadrant, LLQ= left lower quadrant

**Supplementary Table 7: Predictors of MSK Sequelae Over Study Duration in EVD Survivors in Eastern Sierra Leone**

| **MSK Symptoms** | | |
| --- | --- | --- |
| **Characteristic** | **OR [CI]** | **p-value** |
| **Age Categories** |  |  |
| 15-40 y vs <15 y | 4.0 [1.1, 15.4] | .043 |
| >40 y vs <15 y | 3.4 [0.7, 15.3] | .116 |
| 15-40 y vs >40 y | 1.2 [0.4, 3.0] | .763 |
| **Female** | 1.2 [0.8, 1.8] | .305 |
| **Depression** | 15.5 [3.9, 61.2] | <.001 |
| **MSK Signs** | | |
| **Age Categories** |  |  |
| 15-40 y vs <15 y | 0.4 [0.0, 4.1] | .411* |
| >40 y vs <15 y | 0.1 [0.0, 1.4] | .086* |
| 15-40 y vs >40 y | 4.7 [1.6, 14.3] | .006* |
| **Female** | 1.3 [0.9, 2.1] | .209 |
| **Depression** | 1.7 [0.4, 6.6] | .451 |

Age categories, sex, and self-reported depression predict MSK sequelae to varying degrees. Models adjusted for age, sex, and time from discharge to visit. Generalized linear mixed model used for analysis in R Studio. *= significant interaction between variable and days since discharge to visit. Abbreviations: y= years, MSK= musculoskeletal, EVD= Ebola virus disease, OR= odds ratio, CI= confidence interval.

**Supplementary Figure Legends**

**Supplementary Figure 1: Adjusted Odds of MSK Sequelae in EVD Survivors at Enrollment**. Odds ratios adjusted for sex and time from hospital discharge to first visit. 1A: Adjusted odds ratios for MSK sequelae in EVD survivors in 15-40 years and >40 years age groups compared to survivors <15 years of age. 1B: Adjusted odds ratios for MSK sequelae in EVD survivors 15-40 years of age compared to survivors >40 years of age. Statistics performed in R Studio. Graph created in GraphPad Prism. Abbreviations: * = p-value <.05, MSK= musculoskeletal, EVD= Ebola virus disease.

**Supplementary Figure 2: MSK Signs and Symptoms in EVD Survivors More Than Seven Years Since ETU Discharge in Eastern Sierra Leone**. MSK signs and symptoms generally decrease over time. MSK symptoms are reported more in years 5 and 6 than year 4, however these increases were not statistically significant in logistic regression (Year 5 vs Year 4: p= 0.991; Year 6 vs Year 5: p=1.0). Statistics performed in R Studio. Heatmap made in GraphPad Prism. Abbreviations: MSK= musculoskeletal, EVD= Ebola virus disease, ETU= Ebola treatment unit.
