# Supplementary figures and images for: Characterizing Musculoskeletal Sequelae in Ebola Virus Survivors During the 7 Years Since Hospital Discharge in Eastern Sierra Leone
Source: Open Forum Infect Dis. 2025 Mar 8;12(4):ofaf129. doi: 10.1093/ofid/ofaf129 (PMC11952999; doi:10.1093/ofid/ofaf129)

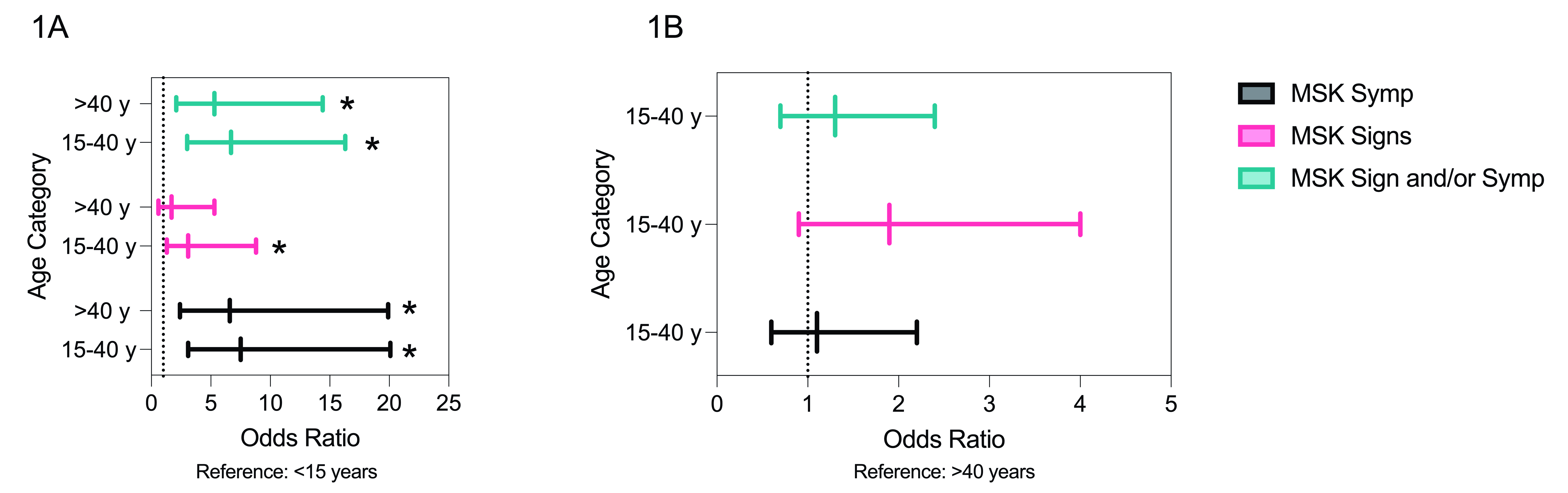

Supplement: ofaf129_Supplementary_Data [file ofaf129_supplementary_data.zip › figure_s1.tiff]

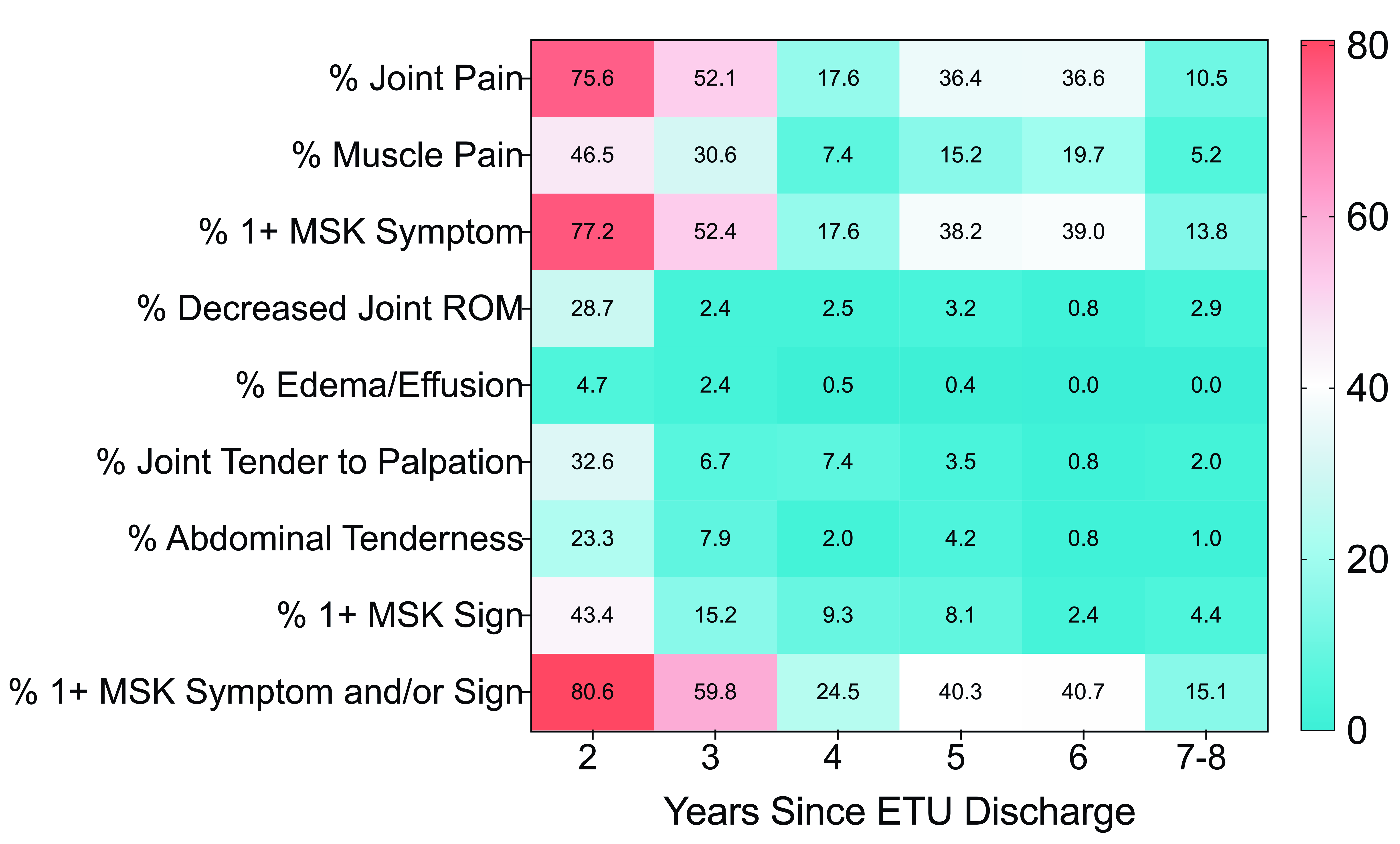

Supplement: ofaf129_Supplementary_Data [file ofaf129_supplementary_data.zip › figure_s2.tiff]
